# Bringing the band back together: eukaryotic microbes in microbial genomics

**DOI:** 10.1099/mgen.0.001652

**Published:** 2026-02-12

**Authors:** Eva Heinz, Fiona L. Henriquez, Virginia M. Howick

**Affiliations:** 1 Strathclyde Institute of Pharmacy and Biomedical Sciences, University of Strathclyde, Glasgow, UK; 2 Department of Civil and Environmental Engineering, University of Strathclyde, Glasgow, UK; 3 School of Biodiversity, One Health & Veterinary Medicine, University of Glasgow, Glasgow, UK

As a group, eukaryotic microbes occupy a unique position in biology, bridging the conceptual space between classical microbiology and complex multicellular systems. Their cellular organization, diverse life cycles and intricate interactions with hosts and different environments challenge known models of microbial function and evolution. Unravelling this complexity is not merely an exercise in classification; it offers fundamentally new perspectives on pathogenicity, symbiosis, adaptation and ecosystem function. From revealing unconventional modes of gene regulation and genome organization to reshaping our understanding of host–microbe interactions and environmental resilience, studies of microbial eukaryotes continue to advance our knowledge of biological principles. As such, eukaryotic microbes provide powerful lenses through which to generate innovative approaches across medicine, agriculture, biotechnology and environmental science.

The recent advances in long-read sequencing and the ever-falling costs of sequencing have given rise to exciting and groundbreaking insights across microbial eukaryotes, which led to the first instalment of a microbial eukaryote-focused conference sponsored by Microbial Genomics. Given their high diversity, different taxonomies and very variable impact on human health, eukaryotic microbes are found across different scientific communities, including parasites, protists, algae and fungi, which we aimed to bring together at the University of Strathclyde, Glasgow, in September 2025.

This deliberate effort led to an exciting programme of talks which reflected the conceptual and technical breadth of microbial eukaryote genomics, with invited speakers highlighting how new genomic approaches are reshaping long-standing biological questions. Dr Maria Rosa Domingo Sananes (Nottingham Trent University, UK) opened the scientific programme by exploring how evolutionary processes can repair disrupted mitotic entry networks, illustrating how system-level thinking can reveal unexpected routes to cellular robustness. Later in the day, Dr Emma Briggs (Newcastle University, UK) demonstrated how single-cell transcriptomics is transforming our understanding of cell cycle progression and developmental heterogeneity in kinetoplastid parasites, offering fine-grained insights that would be inaccessible using bulk approaches. Together, these contributions exemplified how cutting-edge methodologies are enabling deeper interrogation of eukaryotic microbial biology across evolutionary and cellular scales.

The broader programme showcased emerging work on genome architecture, gene gain and loss, host–pathogen interactions and environmental adaptation across diverse taxa, including fungi, protists and parasitic trypanosomatids. Talks on pangenome dynamics in *Aspergillus fumigatus*, genome restructuring driven by sexual reproduction in *Trypanosoma cruzi* and drug-induced changes in fungal pathogenicity highlighted the translational relevance of microbial eukaryote genomics to human health and biotechnology. By bringing these studies into a shared forum, the meeting emphasized common challenges while reinforcing the value of cross-fertilization between research communities that are often siloed by organism or application.

Furthermore, to capture and highlight the research on this fascinating group of organisms, the conference marked the launch of the collection on microbial eukaryotes in Microbial Genomics. The fascinating works so far feature everything from parasites to marine protists, pathogenic fungi and genetics, contributed by scientists from 11 countries spanning Australia, Asia, Africa, Europe, North America and South America, demonstrating the global relevance of these organisms, and we are excited to provide a joint space for this global community. The collection is still open until June 2026, and we are greatly looking forward to your contributions. Following the success of the conference, we are also considering a reinstatement of this cross-community meeting for 2027; keep your eye out for news!

